# Monogenic systemic lupus erythematosus onset in a 13-year-old boy with Noonan like-syndrome: a case report and literature review

**DOI:** 10.1186/s12969-023-00939-z

**Published:** 2024-01-18

**Authors:** Patricia Morán-Álvarez, Alessandra Gianviti, Francesca Diomedi-Camassei, Monia Ginevrino, Fabrizio de Benedetti, Claudia Bracaglia

**Affiliations:** 1 Division of Rheumatology, ERN RITA center, IRCCS Ospedale Pediatrico Bambino Gesù, Rome, Italy; 2 Universidad Alcalá de Henares, Madrid, Spain; 3 Division of Nephrology, IRCCS Ospedale Pediatrico Bambino Gesù, Rome, Italy; 4 Division of Pathology, IRCCS Ospedale Pediatrico Bambino Gesù, Rome, Italy; 5 Laboratory of Medical Genetics, Translational Cytogenomics Reseach Unit, IRCCS Bambino Gesù Children’s Hospital, Rome, Italy

**Keywords:** Systemic lupus erythematosus, Noonan syndrome, Monogenic, Children, Genetic, SHOC2, RAS/MAPK, Zebra bodies

## Abstract

**Background:**

Childhood systemic lupus erythematosus (cSLE) has been considered as a polygenic autoimmune disease; however, a monogenic lupus-like phenotype is emerging with the recent recognition of several related novel high-penetrance genetic variants. RASopathies, a group of disorders caused by mutations in the RAS/MAPK pathway, have been recently described as a cause of monogenic lupus.

**Case presentation:**

We present a 13-year-old boy with Noonan-like syndrome with loose anagen hair who developed a monogenic lupus. The renal biopsy confirmed a class III lupus nephritis and identified the presence of zebra bodies.

**Conclusions:**

RASopathies represent a cause of monogenic lupus. We report a new case of monogenic lupus in a child with Noonan-like syndrome with loose anagen hair. Lupus nephritis which has never been described in this context, may be part of the presentation. The presence of zebra bodies in SLE or RASopathies in unclear, but no other known conditions (Fabry disease or drugs) were identified as the cause of zebra bodies in our patient.

## Background

Childhood systemic lupus erythematosus (cSLE) is a chronic, life-threating, multi-system autoimmune disease diagnosed in children under the age of 18 years [1]. Its prevalence ranges from 3.3 to 24 per 100,000 children [2]. cSLE usually presents a more severe clinical course with a higher frequency of major organ involvement compared to adult-onset [1]. Recent studies suggest that genetic basis may play an important role in cSLE pathogenesis. A less striking sex distribution and well-known genetic conditions with lupus features, such as Aicardi-Goutières syndrome or A20 haploinsufficiency, are some of the evidence which support this fact.

Recent studies have reported that some of the mutations causing RASopathies have also been related to monogenic lupus [3]. One of these conditions is Noonan-like syndrome with loose anagen hair. A dysregulation of RAS signaling due to a recurrent missense variant in *SHOC2* gene is responsible for this syndrome. The activation of the RAS/ MAPK pathway in immune cells leads also to the development of autoimmune disorders such as SLE [4].

We report a monogenic lupus case in a child with Noonan-like syndrome with loose anagen hair.

## Case presentation

A thirteen-year-old boy with a molecularly confirmed diagnosis of Noonan-like syndrome with loose anagen hair (*SHOC2*, c.4 A > G, p.Ser2Gly) was admitted to our hospital with a 2-week-history of polyarthritis, rash, cytopenia, abdominal pain, malaise, and general weakness. He had been evaluated at the emergency department for swollen and painful ankles, associated with abdominal pain, purpuric rash and microhematuria. An initial diagnosis of Henoch-Schönlein Purpura was established. However, due to the persistence of the symptoms he was finally admitted to our rheumatology division for additional evaluation.

**Fig. 1:**
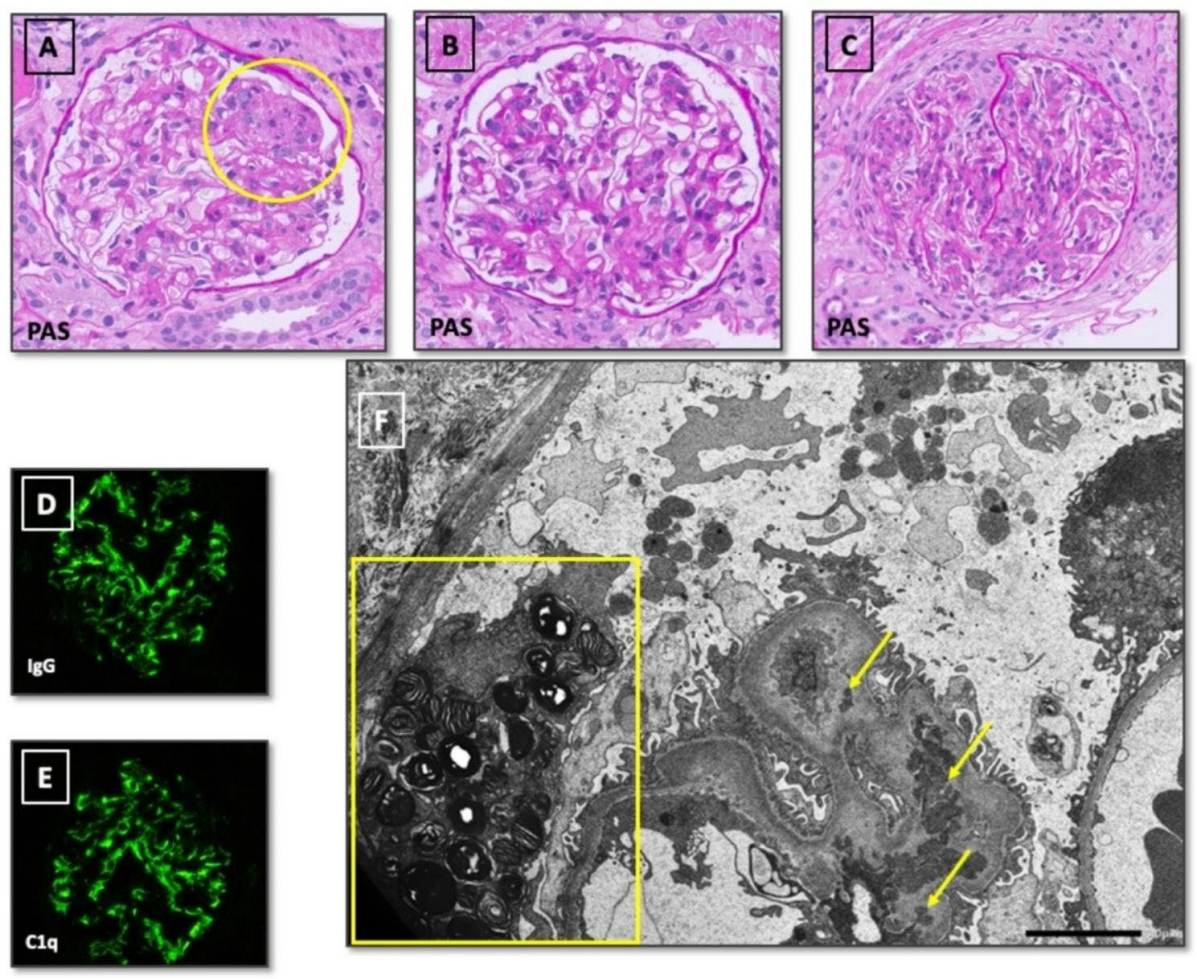
Histology (P.A.S. stain, magnification 40x): renal biopsy showed 14 glomeruli. In < 50%, variable mesangial hypercellularity was present: focal/segmental in **A**, mild in **B** and moderate in **C**. In **A**, a focus of endocapillary proliferation was observed (circle). Immunofluorescence (magnification 40x) showed global positivity for IgG+++ (**D**), IgA++, C3++, C1q+++ (**E**), kappa light chain+++ and lambda light chain+++. IgM were negative. Electron microscopy (**F**, magnification 2500x) highlighted many subendothelial deposits (arrows) and laminated zebra bodies in the cytoplasm of few podocytes (rectangle)

Laboratory tests revealed lymphopenia (lymphocyte count 660/µL), thrombocytopenia (platelet count 129,000/µL), non-hemolytic hypochromic anemia (10,9 g/dL), elevated C reactive protein (CRP) and erythrocyte sedimentation rate (ESR) (9,99 mg/dL and 28 mm/h, respectively). Low levels of complement (C3 73 mg/dL and C4 level 2 mg/dL) were present. Autoantibody screening showed antinuclear antibodies (ANA) with a titer of 1:2560 with a homogeneous pattern, anti-dsDNA antibodies (1:640), anti-SSA/Ro (174 U/mL), and a triple antiphospholipid antibody positivity (IgG anticardiolipin 34 GPLU/mL, IgG anti-B2-glycoprotein 27 U/mL and positive lupus anticoagulant). A chest x-ray and an echocardiogram were remarkable for pleural and pericardial effusion without hemodynamic compromise. The patient had a normal renal function; however, the urinalysis revealed a 770 mg/ 24 h proteinuria with associated microhematuria and leukocyturia. A renal biopsy provided evidence of a class III lupus nephritis according to ISN/RPS 2003 classification (Fig. 1). In addition, enlarged intralysosomal osmiophilic, lamellar and concentric inclusions in podocytes resembling “zebra bodies” were identified at the electron microscopy (Fig. 1F). No congenital cardiac disorders related to Noonan-like syndrome were observed.

A SLE diagnosis, according to EULAR/ACR 2019 classification criteria, was established based on the presence of clinical (lymphopenia, thrombocytopenia, lupus nephritis, arthritis, serositis) and immunological criteria (positive ANA, anti-dsDNA, antiphospholipid antibodies, lupus anticoagulant, and low complement).

The patient started treatment with methylprednisolone pulses, 30 mg/Kg/day for 3 consecutive days, and afterwards with prednisone at 2 mg/Kg/day; associated with hydroxychloroquine (5 mg/Kg/day) and mycophenolate mofetil (600 mg/m^2^/12 h). In addition, a primary thromboprophylaxis with acetylsalicylic acid (100 mg/day) was also started due to the presence of a high-risk antiphospholipid profile—triple positivity. A progressive clinical and biochemical good response was observed.

The parents decided to continue follow-up in another center. During a phone contact one year after discharge, the parents reported no additional health issue while significantly tapering treatment.

## Discussion and conclusions

SLE is a chronic systemic autoimmune disease which leads to inflammation and organ damage caused by immune complex deposition. Classically, cSLE has been considered as a polygenic autoimmune disease; however, monogenic lupus-like phenotypes in children are emerging with the recent recognition of several related novel high-penetrance genetic variants in the last decade [5]. This fact associated with the high degree of concordance among monozygotic twins, supports the relevance of genetic background in the childhood lupus pathogenesis [3, 5, 6].

Currently, mutations in more than 50 different genes related to complement (e.g. C1, C2, C4), type I interferon (e.g. *DNASE, RNASE, TREX, IFIH1, ADAR*), self-tolerance (e.g. *PRKCD, TNFSF13B*) or RAS pathways (e.g. *PTPN11, SOS1, KRAS, SHOC2*) have been considered responsible for the development of monogenic lupus [3].

At least 20 genetic variants involved in the RAS/MAPK pathway function, are included in a group of disorders collectively defined as RASopathies. Facial dysmorphism, cardiac disease, growth hormone deficiency, and cognitive defects are the main clinical features of RASopathies [7]. Pathogenic variant in *SHOC2* gene, a scaffold protein with an up-regulatory function on RAS/MAPK pathway, leads to Noonan-like syndrome with loose anagen hair which is characterized by facial feature and ectodermal abnormalities (hair anomalies and hyperkeratotic skin lesions) as distinguishing aspects [8]. In addition to these manifestations, a link between Noonan-like syndrome and autoimmune disorders (such as SLE, thyroiditis or hepatitis) has been postulated due to the role of RAS on T cell maturation. This fact suggests that abnormal activation of the RAS/MAPK pathway may be a risk condition for the development of autoimmune diseases [4].

We report a 13-year-old-boy with a constitutional *SHOC2* genetic variant (c.4 A > G, p.Ser2Gly) who developed a monogenic lupus. This is similar to what observed in RASopathies and specifically in *SHOC2*-carrier patients, which have a male prevalence and a median age of SLE onset around their teens [3]. These data suggest a distinctive underlying pathway on monogenic lupus pathogenesis compared to the classical SLE.

A total of 12 patients with a Noonan syndrome and an associated SLE diagnosis have been described in the literature to date. Of them, 4 patients carried a constitutional *SHOC2* genetic variant, 2 patients a *KRAS* variant, 1 patient a *PTPN11* variant and in the remaining 5 patients no genetic variant was specified. Our patient represents the fifth *SHOC2*-carrier with a diagnosis of monogenic lupus [3, 9–12].

Musculoskeletal disorders (8/11 patients) are the most common manifestations in patients with RASopathies. Moreover, other lupus clinical manifestations have also been reported: pleuro-pericarditis (7/11 patients), autoimmune cytopenia (6/11 patients) and rash (2/11 patients) [3]. Specifically, in *SHOC2* carriers, serositis was the most frequent clinical manifestation (3/3 patients), followed by cytopenia (2/4 patients), polyarthritis (1/3 patients) and mucocutaneous disorders (1/3 patients). No neuropsychiatric or renal manifestations have been previously reported [9–12]. Our patient onset with thrombocytopenia, lymphopenia, polyarthritis of the large and small joints and serositis (pleural and pericardial effusion), which is similar to those described above. However, a lupus nephritis in *SHOC2* carriers has not been previously reported.

Regarding immunologic parameters, 3 patients with a *SHOC2* variant were positive for ANA and anti-double stranded-DNA (ds-DNA) antibodies, 1 was positive for anti-Smith antibodies (anti-Sm) and one showed low levels of complement - both C3 and C4 [9–12]. Our patient showed ANA, anti-ds-DNA, anti-SSA/Ro positivity with reduced levels of complement. Demographic characteristics, EULAR/ACR 2019 SLE classification criteria, therapeutic strategy and response to the therapy were shown in detail in Table 1.

**Table 1:** Demographic characteristics, clinical and immunological parameters, therapeutic strategy, and response to the therapy

	Simsek-Kiper et al., 2012	Bader-Meunier et al., 2013	Hanaya et al., 2017	Uehara et al., 2018	Currently presented patient
Gender, age at onset (years)	Male	Male, 13	Male, 13	Male, 24	Male, 13
**EULAR/ACR 2019 criteria**					
Fever	NA	NA	NA	NA	YES
Hematologic	Thrombocytopenia	NO	NO	Leukopenia Thrombocytopenia	Leukopenia Lymphopenia Thrombocytopenia
Neuropsychiatric	NA	NO	NO	NO	NO
Mucocutaneous	NA	NO	NO	Malar rash	NO
Pleuritis	NA	Pericarditis	Pericarditis	Pericarditis	Pleuritis and pericarditis
Articular	NA	Polyarthritis	NO	NO	Polyarthritis
Renal	NO	NO	NO	NO	Class III LN Zebra bodies
ANA	NA	1:800	1:640	1:2560	1:2560
Anti-ds-DNA	NA	39 IU	87 IU	> 1:400	1:640
Anti-Smith	NA	Negative	149 U	Negative	Negative
Low complement	NA	NO	YES	C3 42 mg/dL C4 5 mg/dL	C3 73 mg/dL C4 2 mg/dL
aPL and/or LA	NA	NO	NO	YES	IgG aCL, IgG aB2GP, LA
Treatment	NA	HCQ, ASA	GC	GC	HCQ, MMF, GC, ASA
Response	NA	Satisfactory (tapered in 3 m)	Satisfactory (tapered in 20 m)	Satisfactory	Satisfactory

aB2GP, anti-B2-glycoprotein antibodies; aCL, anticardiolipin antibodies; ANA, antinuclear antibodies; aPL, antiphospholipid antibodies; ASA, acetylsalicylic acid; GC, glucocorticoids; HCQ, hydroxychloroquine; LA, lupus anticoagulant; LN, lupus nephritis; NA, not available; m, months; MMF, mycophenolate mofetil

As far as zebra bodies are concerned, this is the first case to date which identify lamellar bodies in a patient with a Noonan-like syndrome and lupus. This renal finding is the result of a blockade of intralysosomal phospholipid catabolism, which culminates in formation of osmiophilic and lamellar inclusions in the lysosome of podocytes. Generally, this condition appears in Fabry disease, but also as a drug-induced condition related to chloroquine or amiodarone [13, 14]. However, our patient was not under any of these conditions. A dysregulation in the RAS/MAPK pathway or SLE have been related with the development of lysosomal storage disorders [15, 16]. Therefore, our findings suggest that these intralysosomal inclusions may also be present in the context of a RASopathy condition.

In conclusion, we report a new case of monogenic lupus in a 13-year-old male patient with Noonan-like syndrome with loose anagen hair. A lupus onset in a patient with dismorphic features should prompt genetic analysis. RASopathies appear to be a rather frequent cause of monogenic lupus. Notably, lupus nephritis which have never been described in this context, may be part of the presentation.

## Data Availability

Not applicable.

## List of abbreviations

ACR: American College of Rheumatology
ANA: Antinuclear antibodies
Anti ds-DNA: anti-double stranded-DNA
cSLE: Childhood systemic lupus erythematosus
CRP: C reactive protein
ESR: Erythrocyte sedimentation rate
EULAR: European League Against Rheumatism
LA: lupus anticoagulant

## References

[1] Harry O, Yasin S, Brunner H (2018). Childhood-onset systemic Lupus Erythematosus: a review and update. J Pediatr.

[2] Kamphuis S, Silverman ED (2010). Prevalence and burden of pediatriconset systemic Lupus Erythematosus. Nat Rev Rheumatol.

[3] Demirkaya E, Sahin S, Romano M, Zhou Q, Aksentijevich I (2020). New Horizons in the genetic etiology of systemic Lupus Erythematosus and Lupus-Like Disease: monogenic lupus and Beyond. J Clin Med.

[4] Quaio CRDC, Carvalho JF, da Silva CA, Bueno C, Brasil AS, Pereira AC, Bertola DR (2012). Autoimmune Disease and multiple autoantibodies in 42 patients with RASopathies. Am J Med Genet Part A.

[5] Alperin JM, Ortiz-Fernández L, Sawalha AH (2018). Monogenic lupus: a developing paradigm of Disease. Front Immunol.

[6] Jeong DC (2018). Monogenic autoimmune Diseases. J Rheumatic Dis.

[7] Ferrero GB, Picco G, Baldassarre G (2012). Transcriptional hallmarks of Noonan syndrome and Noonan-Like syndrome with loose anagen hair. Hum Mutat.

[8] Mazzanti L, Cacciari E, Cicognani A, Bergamaschi R, Scarano E, Forabosco A (2003). Noonan-Like syndrome with loose anagen hair: a new syndrome?. Am J Med Genet A.

[9] Sim¸sek-Kiper P, Alanay Y, Gülhan B, Lissewski C, Türkyılmaz D, Alehan D, Çetin M, Utine GE, Zenker M (2013). Boduro˘ Glu, K. Clinical and molecular analysis of RASopathies in a group of Turkish patients. Clin Genet.

[10] Bader-Meunier B, Cavé H, Jeremiah N, Magerus A, Lanzarotti N, Rieux-Laucat F, Cormier-Daire V (2013). Are RASopathies new monogenic predisposing conditions to the development of systemic Lupus Erythematosus? Case report and systematic review of the literature. Semin Arthritis Rheum.

[11] Hanaya A, Miyamae T, Kishi T, Sahara M, Tani Y, Yamanaka H, Nagata S (2017). Systemic Lupus Erythematosus associated with RASopathy. Mod Rheumatol Case Rep.

[12] Uehara T, Hosogaya N, Matsuo N, Kosaki K (2018). Systemic Lupus Erythematosus in a patient with Noonan syndrome-like disorder with loose anagen hair 1: more than a chance association. Am J Med Genet Part A.

[13] Huang X, Zhang R (2020). Zebra bodies in the kidney. N Engl J Med.

[14] de Menezes Neves PDM, Machado JR, Custódio FB (2017). Ultrastructural deposits appearing as zebra bodies in renal biopsy: fabry Disease?- comparative case reports. BMC Nephrol.

[15] Rebiai R, Givogri MI, Gowrishankar S, Cologna SM, Alford ST, Bongarzone ER (2021). Synaptic function and dysfunction in lysosomal Storage Diseases. Front Cell Neurosci.

[16] Kadosawa K, Morikawa T, Konishi Y (2021). Zebra bodies without fabry Disease or hydroxychloroquine. Clin Exp Nephrol.

